# Safety and efficacy of targeting CD138 with a chimeric antigen receptor for the treatment of multiple myeloma

**DOI:** 10.18632/oncotarget.26792

**Published:** 2019-03-22

**Authors:** Chuang Sun, Aruna Mahendravada, Brandon Ballard, Brandon Kale, Carlos Ramos, John West, Todd Maguire, Katie McKay, Eben Lichtman, Sascha Tuchman, Gianpietro Dotti, Barbara Savoldo

**Affiliations:** ^1^ Lineberger Comprehensive Cancer Center, University of North Carolina at Chapel Hill, Chapel Hill, NC, USA; ^2^ Center for Cell and Gene Therapy, Baylor College of Medicine, The Methodist Hospital and Texas Children's Hospital, Houston, TX, USA; ^3^ Department of Medicine, Baylor College of Medicine, Houston, TX, USA; ^4^ UNC Lineberger Advanced Cellular Therapeutics Facility, University of North Carolina at Chapel Hill, Chapel Hill, NC, USA; ^5^ Division of Hematology/Oncology, Department of Medicine, University of North Carolina at Chapel Hill, Chapel Hill, NC, USA; ^6^ Department of Microbiology and Immunology, University of North Carolina at Chapel Hill, Chapel Hill, NC, USA; ^7^ Department of Pediatrics, University of North Carolina at Chapel Hill, Chapel Hill, NC, USA

**Keywords:** chimeric antigen receptor, adoptive cell transfer, multiple myeloma, T cells immunotherapy, syndecan-1

## Abstract

After unprecedented successes in B-cell malignancies, chimeric antigen receptor T cells have recently been investigated for the treatment of multiple myeloma. Chimeric antigen receptor targeting T cells B-cell maturation antigen (BCMA) on malignant plasma cells have led to impressive clinical responses in recent trials. However, BCMA-negative relapses have been observed, supporting the need for complementary treatment strategies. Here, we explored the feasibility of targeting CD138 (syndecan-1), a surface marker expressed on both normal and malignant plasma cells. We showed that T cells from both healthy donors and from multiple myeloma patients, when transduced with a CD138-specific chimeric antigen receptor, can eliminate tumor cell lines and primary myeloma cells both in vitro and in vivo. CD138 is also expressed by putative myeloma stem cells identified by Hoechst staining, and these cells can be eliminated by CD138-specific chimeric antigen receptor T cells. Preclinical analyses did not identify any on target off tumor cytotoxicity against normal epithelial or endothelial cells, further supporting the rationale for the use of adoptively transferred CD138-specific chimeric antigen receptor T cells for the treatment of patients with relapsed/refractory multiple myeloma.

## INTRODUCTION

Multiple myeloma (MM) accounts for approximately 17% of hematologic malignancies and 2% of all cancers in the United States [1–3]. Although the survival of MM patients has improved significantly over the past 15 years, most patients will continue to experience repeated relapses, with subsequent remissions becoming increasingly short, and will eventually succumb to refractory disease [4]. Malignant plasma cells are susceptible to cell-mediated immune targeting as evidenced by the graft-versus-myeloma effect occurring after allogeneic hematopoietic stem cell transplantation [5]. Growing evidence also suggests that altered cellular immunity plays a role in the development of MM. Furthermore, targeting MM-associated surface antigens with monoclonal antibodies such as daratumumab [6] and elotuzumab [7], specific for CD38 and SLAMF7 (CS1), respectively, has proven to be a highly effective approach. Given evidence to support T-cell dysfunction in MM, the combination of antibody-based therapies with immune modulators [8] also seems especially well-suited for this disease [9–11].

The clinical success of chimeric antigen receptor (CAR) T cells (CAR-Ts) in B-cell malignancies has reinvigorated the interest in developing CAR-Ts for MM. CAR molecules allow antibody mediated recognition of a targeted antigen to be combined with the cytotoxic and proliferative capacity of effector T cells, thereby overcoming several barriers to effective T cell responses, including the downregulation of MHC or costimulatory molecules and dysfunctional TCR signaling [12]. CD19-specific CAR-Ts and κ-light chain specific CAR-Ts have been tested in MM with the goal of targeting plasma cell precursors, and have led to some clinical responses [13]. The most compelling clinical responses have been obtained with CAR-Ts targeting the B-cell maturation antigen (BCMA), with overall response rates over 80% in recent trials [14]. However, patients treated with BCMA-specific CAR-Ts can relapse due to the emergence of tumor cells with low or absent BCMA expression [14]. Durable responses to CAR-T therapy for such patients will therefore require the targeting of additional surface antigens.

CD138 (syndecan-1) is a member of the syndecan family involved in cell-cell and cell-matrix interactions [15]. CD138 expression is a hallmark of both normal plasma cells and of MM tumor cells [15]. Importantly, CD138 appears to be involved in carcinogenesis, specifically in cell proliferation, angiogenesis, tumor invasion, and metastasis, making it an attractive target for CAR-T therapy. Indeed these features should prevent the occurrence of malignant cells lacking CD138 expression [16]. The antibody-drug conjugate BT062 (Biotest AG, Dreieich, Germany), comprising an anti-CD138 chimerized antibody conjugated to the cytotoxic compound, maytansinoid, has been evaluated both as monotherapy, and in combination with either lenalidomide or pomalidomide and low-dose dexamethasone, and was shown to have an acceptable safety profile with preliminary evidence of activity in patients with relapsed/refractory MM [17–19]. The most common adverse events reported were diarrhea, fatigue, and nausea. CD138-specific CAR-Ts (CD138.CAR-Ts) have previously been tested in 5 patients with MM, also with an acceptable safety profile, and with some evidences of disease stabilization [20]. A remarkable response was also observed in a MM patient with extramedullary involvement [21]. Despite reaching initial clinical evaluation in this trial, the safety and efficacy of targeting CD138 with a CAR has not been explored in depth at the preclinical level. We have developed a CD138-specific CAR (CD138.CAR) using the single chain variable fragment (scFv) sequence derived from the BT062 chimeric antibody and have optimized the structure of this CAR to achieve optimal antitumor efficacy. Our preclinical assessment shows that the CD138.CAR-Ts we have developed display significant anti-MM activity both *in vitro* and *in vivo*, and are not likely to cause unacceptable on-target, off-tumor toxicity.

## RESULTS

### CD138.CARs are expressed by T cells from healthy donors

**Figure 1:**
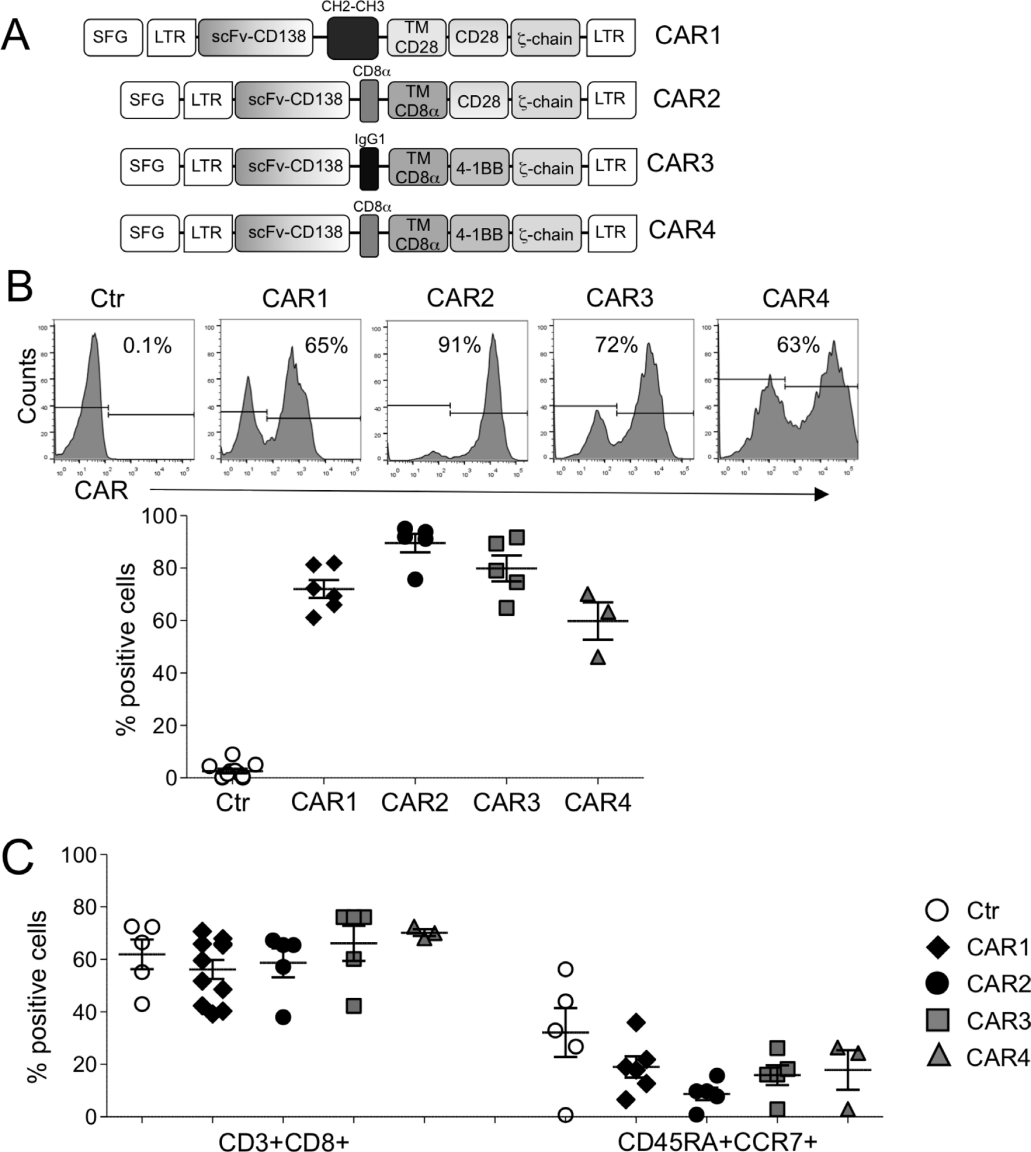
Characterization of CD138.CAR-Ts (**A**) shows the schema of the CD138.CAR retroviral constructs (named CAR1, CAR2, CAR3 and CAR4) used to transduce activated T cells. (**B**) shows CD138.CAR expression evaluated by flow cytometry in control T cells (Ctr-Ts) and in T cells transduced with the four different CD138.CAR constructs. Upper panels are from one representative donor and lower graph shows cumulative data (*n* = 3-6). (**C**) shows the frequency of CD8 and and central memory subsets (CD45RA^+^CCR7^+^) gated on CD3^+^ cells for Ctr-Ts and CD138.CAR-Ts generated from healthy donors (*n* = 3-6).

We have generated four CD138.CAR constructs, each encoding an identical scFv specific for CD138, but different hinges (IgG1.CH2.CH3, IgG1, CD8α), transmembrane domains (CD28, CD8α), and costimulatory endodomains (CD28, 4-1BB); these are referred to as CAR1, CAR2, CAR3, and CAR4 (Figure 1A). Expression of CARs was measured using flow cytometry with a Fab-specific Ab. The transduction efficiency of the various CD138.CARs in blood mononuclear cells obtained from healthy individuals was consistently ≥50% (Figure 1B). The CD138.CAR-Ts expanded *in vitro* and, among the four engineered CARs, there were no significant differences in the composition of CD4^+^ versus CD8^+^ T cells or central/effector memory T cells (Figure 1C).

### CD138.CAR-Ts target CD138^+^ MM cell lines

**Figure 2:**
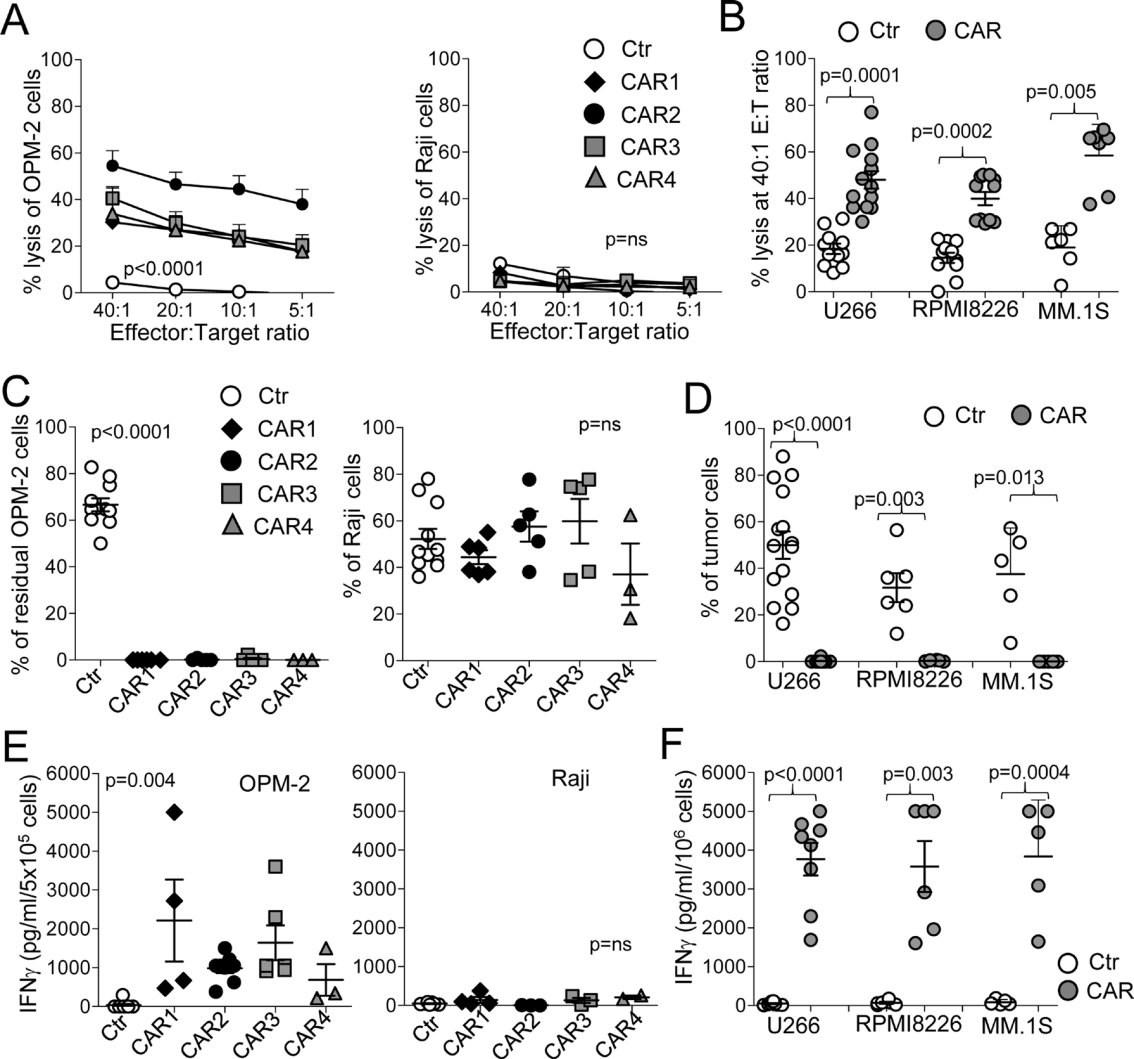
CD138.CAR-Ts specifically lyse CD138^+^ target cells (**A**) shows the results of standard ^51^Cr release assays for CD138^+^ cells (OPM-2 cells left panel) or CD138^−^ tumor cells (Raji, right panel), at the indicted T cell (effector) to tumor cell (E:T) ratio. Symbols represent the mean ± SEM of CD138.CAR-Ts generated from 5 healthy donors (*p <* 0.0001, one-way ANOVA). (**B**) shows results of standard ^51^Cr release assays against other three CD138^+^ MM cell lines (U266, RPMI, MM.1S cells), at the 20:1 E:T ratio for Ctr-Ts or CD138.CAR-Ts (CAR1, CAR2, CAR3, and CAR4 are combined as no differences were observed between each CAR; 1-2 donors/each CAR). Each symbol represents a donor and the lines represent the mean and SEM for the groups. Shown are the p values of CD138.CAR-Ts vs Ctr-Ts against each cell lines using a two-way paired *t*-test. (**C**) shows the percentage of residual tumor cells for CD138^+^ cells (OPM-2, left panel) or CD138^−^ cells (Raji, right panel) co-cultured at 1:1 ratio with Ctr-Ts, or CD138.CAR-Ts. T cells and tumor cells were quantified by flow cytometry (*p <* 0.0001, one-way ANOVA). (**D**) shows the percentage of residual tumor cells using other CD138^+^ MM cell lines (U266, RPMI, MM.1S cells), in co-cultures with Ctr-Ts or CD138.CAR-Ts at 1:1 ratio. Shown are the p values of CD138.CAR-Ts (CAR1, CAR2, CAR3, and CAR4 are combined as no differences were observed between each CAR 1-2 donors for each CAR) vs Ctr-Ts against each cell lines using a two-way paired *t*-test. (**E**) shows the quantification of IFNγ released in the supernatant by Ctr-Ts or CD138.CAR-Ts co-cultured for 24 hrs with CD138^+^ cells (OPM-2, left panel) or CD138^−^ cells (Raji, right panel) at a 1:1 ratio, and quantified by CBA assays (*p* = 0.004, one-way ANOVA). (**F**) shows the quantification of IFNγ released in the supernatant for three additional CD138^+^ cell lines (U266, RPMI, MM.1S cells) by control T cells or by CD138.CAR-Ts (1–3 donors for each CAR). Shown are *p* value, paired *t*-test. *p* = ns indicates non-significant differences.

To ensure that CD138.CAR-Ts targeted CD138^+^ MM cells, we used both standard 5-hour ^51^Cr release assays and 3 - 5 day co-culture assays. All CD138.CAR-Ts generated from healthy donors, irrespective of the CAR construct, lysed the CD138^+^ MM cell lines OPM-2, U266-B1, RPMI-8226, and MM.1S, at a significantly higher rate as compared to control T-cells (Ctr-Ts), while leaving CD138^−^ targets (Raji) unaffected (Figure 2A, 2B). In the absence of cytokines, we then co-cultured CD138.CAR-Ts and Ctr-Ts with the CD138^+^ MM cell lines OPM-2, U266-B1, RPMI-8226, and MM.1S, or the CD138^−^ tumor cells, Raji. Residual tumor cells were measured via flow cytometry analysis at day 3 - 5 of the co-culture. All CD138.CAR-Ts completely eliminated CD138^+^ tumor cells, while tumor cells overgrew in cultures with Ctr-Ts (Figure 2C, 2D and Supplementary Figure 1A). No activity of CD138.CAR-Ts was observed against CD138^−^ tumor cells. Analysis of co-culture supernatants collected after 24 hours showed the presence of Th1 cytokines when CD138.CAR-Ts were co-cultured with CD138^+^ tumor cells (Figure 2E, 2F and Supplementary Figure 1B).

### Lack of activity by CD138.CAR-Ts against normal epithelial and endothelial cells

**Figure 3:**
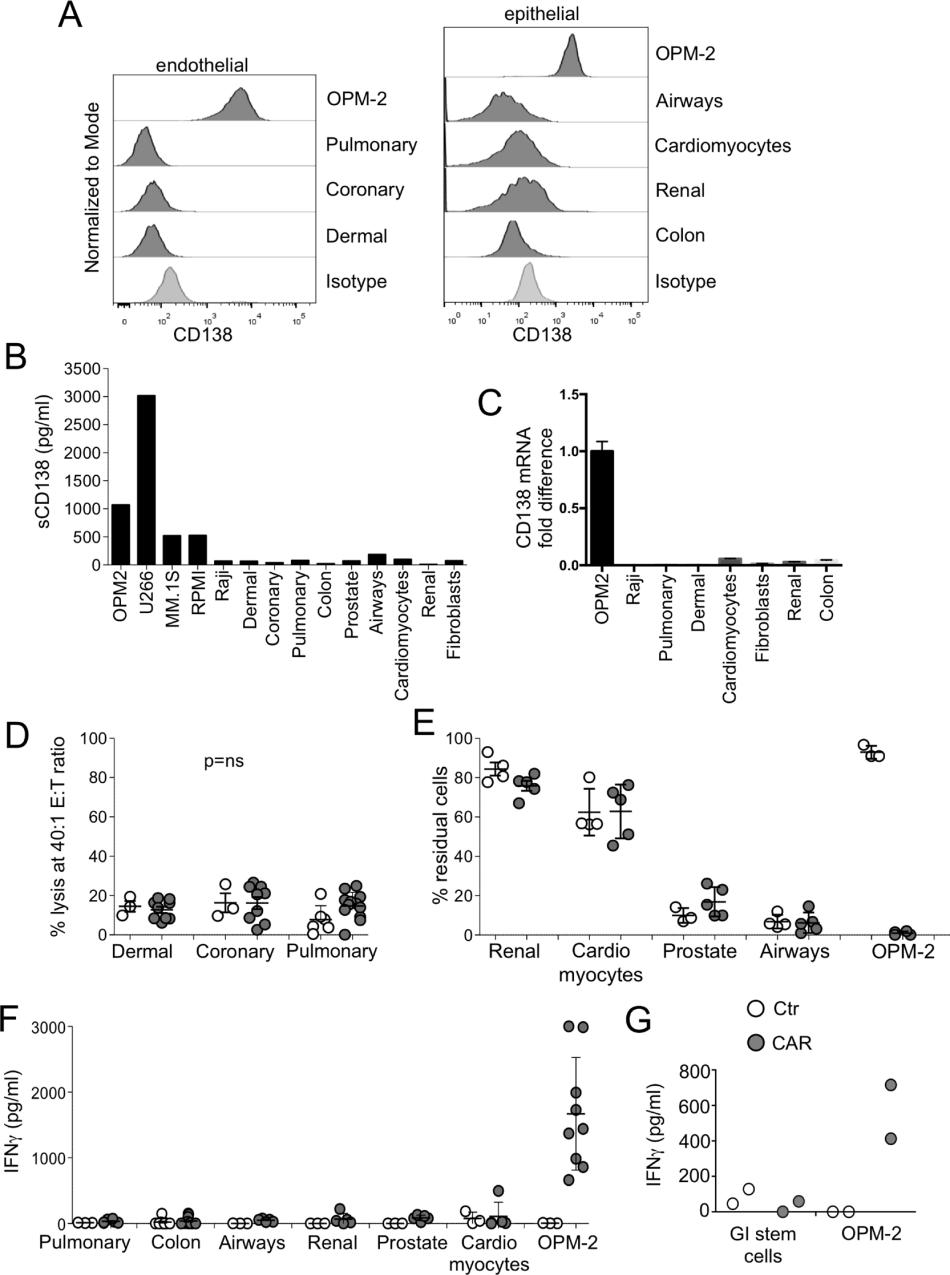
Safety of CD138.CAR-Ts (**A**) shows the expression of the CD138 by commercially available endothelial (right) or epithelial cells (left). Isotype control (of dermal cells for endothelial cells and colon cells for epithelial cells) and CD138 expression of OPM-2 cells are also included. (**B**) shows the evaluation of soluble CD138 in the supernatant of cultured cells using a specific ELISA assay. (**C**) shows the level of CD138 mRNA of cultured cells using qPCR. Data are shown as fold change as compared to the negative control (Raji). (**D**) shows the results of a standard ^51^Cr release assay of endothelial cells at the 40:1 E:T ratio by Ctr-Ts or CD138.CAR-Ts (CAR2, 3 and 4). Each symbol represents a donor and the lines represent the mean and SEM for the groups (Shown are *p* ns value, paired *t*-test.). (**E**) shows the evaluation of residual cells (CD138^+^ tumor cells, OPM-2 or commercially available epithelial or endothelial cells) when co-cultured at 1:1 ratio with Ctr-Ts or CD138.CAR-Ts (CAR 2 and 3 generated from healthy donors). Cells were quantified by flow cytometry. Each symbol represents a donor and the lines represent the mean and SEM for the groups. (**F**) shows the quantification of IFNγ released by Ctr-Ts or CD138.CAR-Ts co-cultured for 24 hrs with OPM-2 cells (positive CD138 control) or commercially available normal cells, and quantified by CBA assays (*p* = ns, one-way ANOVA). (**G**) shows the production of IFNγ in the supernatant of OPM-2 cells or differentiated adult gastrointestinal (GI) stem cells co-cultured with Ctr-Ts or CD138.CAR-Ts (CAR3).

CD138 has been reported to be expressed, based on IHC analysis, on the basolateral surface of some mature epithelial cells, endothelial cells, and vascular smooth muscle cells [15]. With the same antibody used to evaluate CD138 expression by for flow cytometry in MM cell lines, we also assessed commercially available endothelial and epithelial cells for expression of CD138. All tested endothelial and epithelial cells were found to be negative for surface expression of CD138 by flow cytometry (Figure 3A). No measurable soluble CD138 was found in the cell supernatants of these cells (Figure 3B). Because CAR T cells are typically be infused in the context of lymphodepleting chemotherapy, we investigated whether such therapy could induce CD138 expression in endothelial cells. We found that neither drugs frequently used in the treatment of MM (e.g. melphalan, dexamethasone, and cyclophosphamide) nor drugs used as lymphodepleting agents prior to CAR-T administration (cyclophosphamide or fludarabine) upregulated the CD138 molecule (Supplementary Figure 2A). Finally, using a specific qPCR assay, we showed that CD138 mRNA transcript was expressed at insignificant levels in endothelial and epithelial cells (Figure 3C). When evaluated in cytotoxicity assays, both CD138.CAR-Ts and Ctr-Ts exhibited negligible lysis of endothelial and epithelial cells (Figure 3D and Supplementary Figure 2B). Similarly, no elimination of endothelial or epithelial cells was observed in co-culture assays (Figure 3E and Supplementary Figure 2C) by CD138.CAR-Ts or Ctr-Ts, and there was no significant release of IFNγ (Figure 3F). We further confirmed the safety of CD138.CAR-Ts on gastrointestinal (GI) cells by co-culturing them with undifferentiated and differentiated adult GI stem cells. No significant amount of IFNγ was detected when CD138.CAR-Ts were exposed to differentiated (Figure 3G) or undifferentiated/spontaneously differentiated (Supplementary Figure 2D) GI stem cells.

### CD138.CAR-Ts can be generated from MM patients and target autologous CD138^+^ MM cells and putative MM cancer stem cells

**Figure 4:**
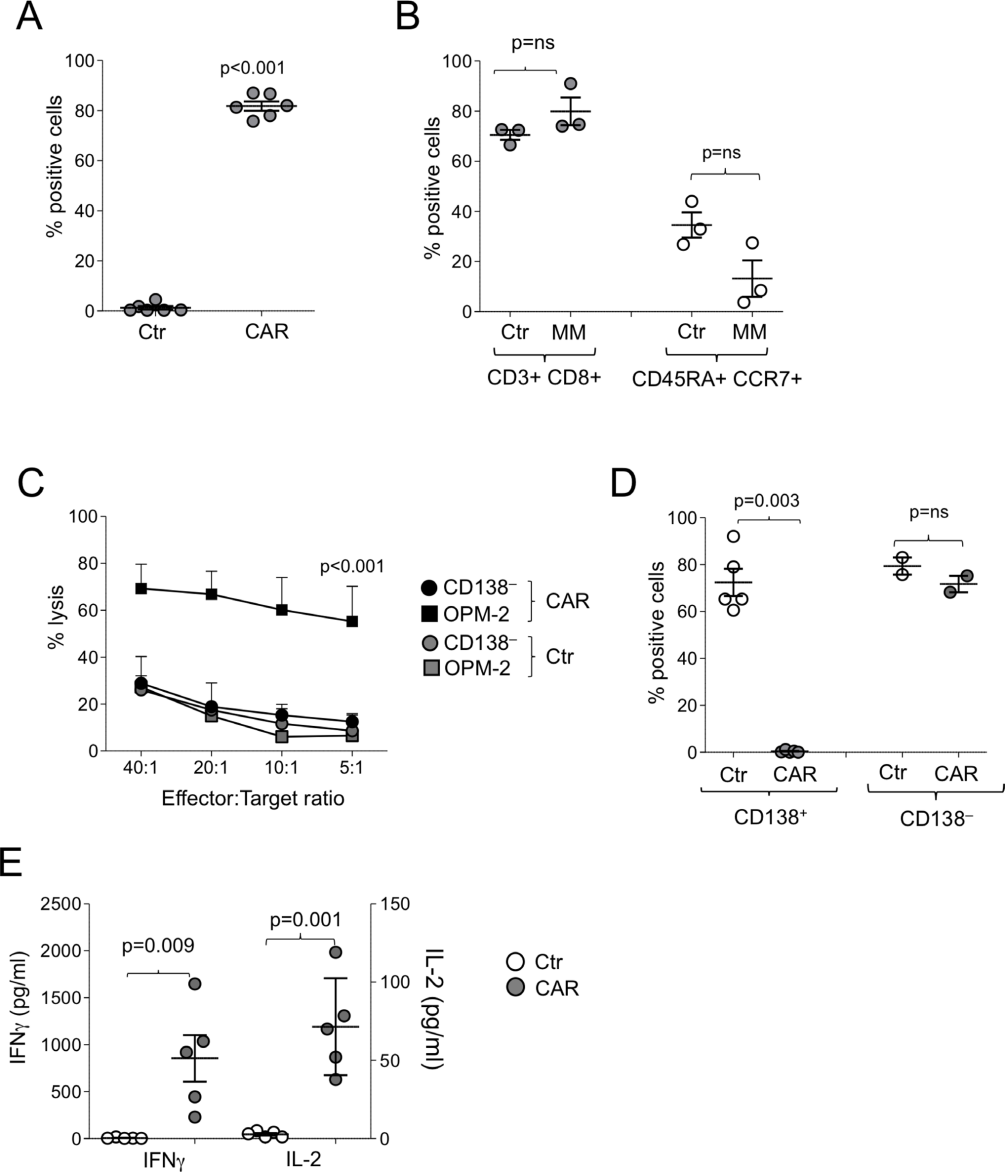
Function of CD138.CAR-Ts generated from MM patients (**A**) shows the expression of the CAR.CD138 (CAR2) evaluated by flow cytometry in Ctr-Ts or CD138.CAR-Ts generated from 6 MM patients (*p <* 0.001, two-way paired t-test). (**B**) shows the immunophenotype of Ctr-Ts or CD138.CAR-Ts generated from MM pateints. Shown are *p* = ns value, paired *t*-test. (**C**) shows the results of a standard ^51^Cr release assay against CD138^+^ (OPM-2) or CD138^−^ (Raji) tumor cell lines, at the indicted T cell to tumor cell ratio. Symbols represent the mean ± SEM of the Ts generated from 4 MM patients (*p <* 0.001, one-way ANOVA). (**D**) shows the percentage of tumor cells in experiments in which CD138^+^ cells (OPM-2, left) or CD138^−^ cells (Raji, right) were co-cultured at 1:1 ratio with Ctr-Ts or CD138.CAR-Ts. T cells and tumor cells were quantified by flow cytometry (*p* = 0.003, and *p* = ns, two-way paired *t*-test). (**E**) shows the quantification of IFNγ and IL-2 released by Ctr-Ts and CD138.CAR-Ts co-cultured for 24 hrs with CD138^+^ cells (OPM-2) at 1:1 ratio and quantified by CBA assays (*p* = 0.009 for IFNγ and *p* = 0.001 for IL-2, two-way paired *t*-test).

We generated CD138.CAR-Ts from MM patients and tested them against MM cells collected from the same patients. Due to the limited number of available T cells and tumor cells from MM patients, we could not compare all constructs, and thus elected to transduce T cells only with CAR1. We reasoned that while other versions of CD138.CAR (e.g. CAR2 and CAR3) behaved superiorly, CAR1 activity on primary cells suggested that even a suboptimal CAR produces appropriate antitumor activity. Transduction efficiency of CAR-Ts (Figure 4A) and immune-phenotype composition mirrored that of healthy donors (Figure 4B). We also found a similar pattern of target specificity using both ^51^Cr release assays (Figure 4C) and co-culture assays (Figure 4D). CD138.CAR-Ts released IFNγ and IL-2 in response to CD138^+^ MM cell lines (Figure 4E).

**Figure 5:**
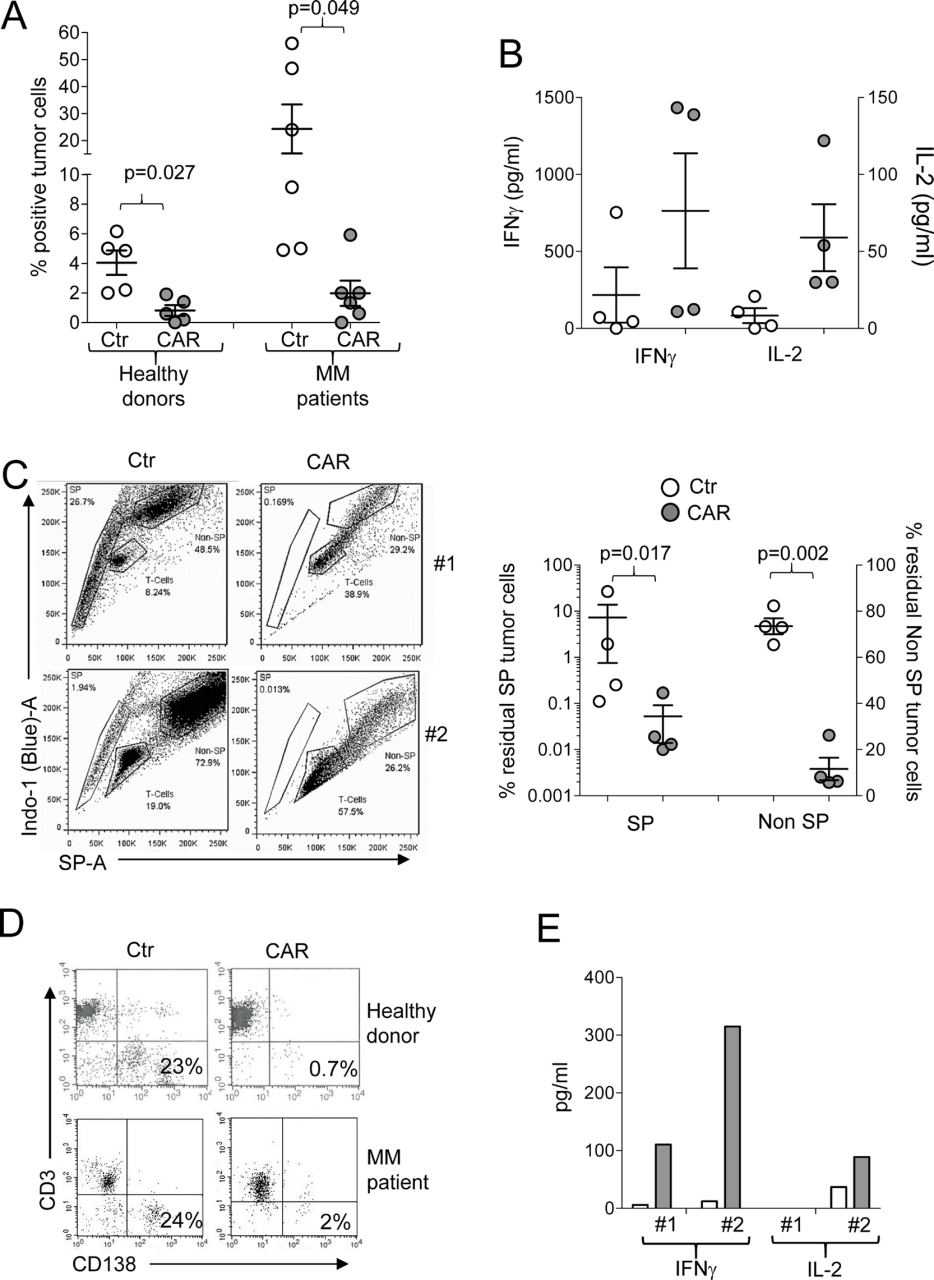
Targeting of primary MM tumors (**A**) shows the percentage of residual MM tumor cells in experiments in which primary MM cells were co-cultured with autologous (MM patients derived) or allogeneic (healthy donor derived) Ctr-Ts and CD138.CAR-Ts. T cells and malignant plasma cells were quantified by flow cytometry. Each symbol represents a donor and the lines represent the mean and SEM for the groups. Shown are the *p* values using a two-way paired *t*-test. (**B**) shows the quantification of IFNγ, and IL-2 released in the supernatant by Ctr-Ts or CD138.CAR-Ts generated from MM patients co-cultured for 24 hrs with autologous malignant plasma cells, and quantified by CBA assays. (**C**) shows the flow plots for two representative donors of Ctr-Ts or CD138.CAR-Ts co-cultured with CD138^+^ RPMI MM cell line. SP cells, non-SP cells and T cells at the end of the 3 days co-culture are shown. The graph on the right panel summaries the residual SP cells and non-SP cells in co-culture with CD138.CAR-Ts obtained from healthy donors (*n* = 2) and MM patients (*n* = 2). Shown are *p* value of two-way paired *t*-test. (**D**) shows the flow plots for two representative donors of Ctr-Ts or CD138.CAR-Ts co-cultured with SP cells sorted from the RPMI MM cell line. (**E**) shows the quantification of IFNγ and IL-2 released in the supernatant of Ctr-Ts or CD138.CAR-Ts from 2 representative donors co-cultured with sorted SP cells, and quantified by CBA-assay.

When patient bone marrow aspirates infiltrated with malignant plasma cells were available, we tested CD138.CAR-Ts in an autologous setting. CD138^+^ cells were isolated from the bone marrow using microbeads and were co-cultured with CD138.CAR-Ts generated from the patients (autologous) or from healthy donors (allogeneic). Both allogeneic and autologous CD138.CAR-Ts eliminated CD138^+^ myeloma cells (Figure 5A) and released Th1 cytokines (Figure 5B). Finally, to assess whether CD138.CAR-Ts can target putative MM stem cells, we used Hoechst staining to identify side-population (SP) cells in MM tumor cell lines and in bone marrow samples as a surrogate functional assay for cancer stem cells [22–25]. We detected SP cells in the RPMI-8221 MM tumor cell line and thus co-cultured Ctr-Ts with RPMI-8226 MM cell line and found that both non-SP and SP cells were still present (73% ± 3.5% and 7% ± 6%, respectively), while CD138.CAR-Ts eliminated better both cell subsets (11% ± 5% and 0.05% ± 0.03%, respectively) (Figure 5C). We also directly sorted SP cells from RPMI-8226 and cultured those with either Ctr-Ts or CD138.CAR-Ts. SP cells from RPMI-8226 were eliminated only in the presence of CD138.CAR-Ts, and supernatant from these co-cultures showed the presence of Th1 cytokines (Figure 5D).

### CAR.CD138-T cells are functional in xenograft models

**Figure 6:**
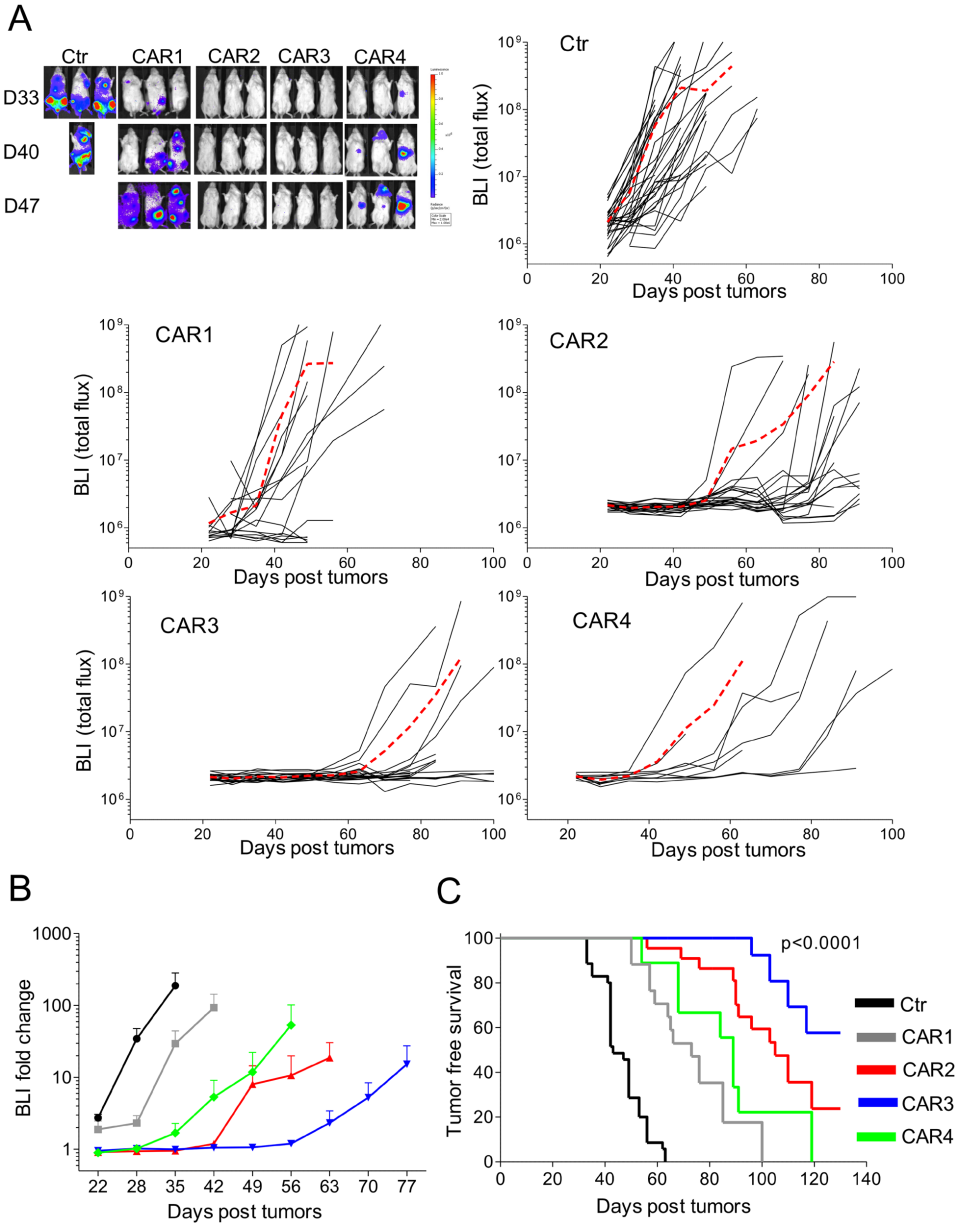
*In vivo* antitumor activity of CD138.CAR-Ts NSG mice were engrafted with CD138^+^ OPM-2 cell line labeled with firefly (FF)-luciferase (4 × 10^6^), and treated 2 weeks later with Ctr-Ts or CD138.CAR-Ts (1 × 10^7^). (**A**) shows the bioluminescence signals (BLI) of Ctr-Ts and CD138.CAR-Ts. Each line shows the BLI for each mouse and the red dashed line the average of the BLI intensity for each group. (**B**) shows the fold expansion of BLI as compared to day 14 (day of T cell injection) summarized as average (± SEM). (**C**) shows the Kaplan-Meier survival curve [Ctr-Ts = 34 mice (T cells from a total of 7 donors); CAR1 = 17 mice (3 donors); CAR2 = 22 mice (5 donors); CAR3=18 mice (4 donors); CAR4 = 10 mice (2 donors)].

We assessed the *in vivo* activity of CD138.CAR-Ts in an NSG mouse-model of MM. All CD138.CARs were tested *in vivo* despite their similar activity *in vitro*. CD138.CAR-Ts or Ctr-Ts were infused i.v. in mice engrafted 2 weeks earlier with OPM-2 MM cells transduced to express firefly luciferase. We selected the OPM-2 as the MM target *in vivo* since this tumor cell line lacks the expression of co-stimulatory molecules, while the other tumor cell lines retain the expression of either CD80 or CD86 (Supplementary Figure 3A). Tumors, monitored using *in vivo* bioluminescence (IVIS), grew rapidly in mice that received Ctr-Ts. In contrast, tumor growth was significantly delayed in mice receiving CD138.CAR-Ts (*p* = 0.04). However, CAR3-expressing T cells showed superior control of tumor growth (Figure 6A and 6B) and better overall survival (*p <* 0.0001) (Figure 6C). Specifically, tumor free survival was superior for mice receiving T cells expressing CAR3 (*p* = 0.026 as compared to CAR2). In addition, more mice treated with CAR3 (8 of 9 analyzed mice) had detectable T cells in the peripheral blood than mice treated with CAR2 (2 of 8) at the end of experiments, suggesting a better persistence of the former (Supplementary Figure 3B). Of note, we confirmed that tumor isolated from mice with recurrent disease retained expression of CD138 (Supplementary Figure 3C), excluding the possibility of antigen escape. To further demonstrate the clinical applicability of the proposed approach, we have generated a clinical grade PG13 retroviral packaging cell line and produced clinical grade supernatant encoding CAR3. As shown in Supplementary Figure 4, in the validation runs performed under Good Manufacture Practice (GMP), CD138.CAR-Ts maintained similar characteristics to those generated in preclinical experiments, including transduction efficiency, immune phenotype, expansion and cytotoxic activity. These data ensure the clinical feasibility of the proposed strategy in patients with MM.

## DISCUSSION

CAR T cell-based therapy represents an appealing approach for MM, and initial clinical responses in patients treated with BCMA-specific CAR-Ts are encouraging [14]. However, relapse due to antigen-escape remains a challenge, and a comprehensive approach to CAR-T treatment in MM will thus necessitate the ability to target additional MM-associated surface antigens. Here we show that CD138 may represent a safe and effective target for CAR-T-based therapy of MM.

MM is characterized by genetic and phenotypic heterogeneities due to the presence of multiple subclones as the disease progresses [26]. This intrinsic heterogeneity mandates the need to explore additional targets in MM which will likely benefit from combination with BCMA-specific CAR-Ts to prevent tumor escape. Previous clinical experience with monoclonal antibodies can guide antigen selection with the goal of anticipating or preventing life-threatening toxicities when the same molecules are targeted via CAR T cells. For example, the CD38 and CS1 antigens, currently targeted with daratumumab and elotuzumab, respectively, in patients with relapsed/refractory MM, have been proposed for CAR-T-based therapy in MM, and are undergoing pre-clinical assessment [27–30]. The rationale for other targets that have not been previously tested using monoclonal antibodies, such as the isoform variant 6 (CD44v6) [31, 32], CD70 [33–35], and APRIL [36] mainly draws from their expression in a significant fraction of MM samples. However, each of these antigens not uniquely expressed by MM cells or plasma cells, and potential “on target off tumor” effects in patients will require careful assessment. In line with the goal of targeting MM-associated antigens clinically validated with monoclonal antibodies, we have elected to assess the CD138 molecule.

The CD138 antibody-drug conjugate (BT062), when used in patients with MM, was in general well tolerated, although mucositis, stomatitis, and diarrhea had occurred in some patients [17–19]. A group at the Chinese PLA General Hospital treated 5 MM patients with a CD138.CAR encoding 4-1BB, and showed some *in vivo* persistence of the CAR-Ts, and clinical responses in the absence of toxicity [20]. Although encouraging, these results must be interpreted with caution, due to the small sample size of the patient's cohort and variability in responses. Here, we have provided further preclinical studies to support the safety and efficacy of targeting CD138. We observed that the CD138.CAR generated from the BT062 mAb, can be successfully expressed by autologous T cells and promote anti-MM activity *in vitro* and *in vivo* in a xenograft mouse model. The CD138.CAR appears to be unaffected by soluble CD138, which may act as a decoy for CAR-Ts, because efficient killing occurs in co-culture with tumor cells lines that produce large amount of soluble CD138. Shedding of the antigen is not unique to CD138, and other CARs have been shown to retain their killing properties even in the presence of soluble antigen [37, 38]. Having systematically studied the other components (hinge, transmembrane domain and costimulation) of the CAR, we have also identified that, for the CD138.CAR, the combination of a short IgG1 hinge and the 4-1BB co-stimulatory domain has superior *in vivo* efficacy despite similar activity *in vitro*, further underscoring the importance of careful and systematic analysis when constructing a new CAR.

MM is a malignancy in which putative cancer stem cells may play a critical role in causing tumor recurrence and drug resistance [39]. Targeting CD19 and the light-chain of human immunoglobulins with CAR-Ts represent strategies to eliminate primitive B-cell precursors that may act as a reservoir of differentiated malignant plasma cells [13, 40, 41]. We used a functional Hoechst SP cell staining, previously validated to identify more primitive progenitors of hematopoietic stem cells and tumor cells [22, 24], as a surrogate marker of putative cancer stem cells in MM. We observed that SP cells express CD138, and can be effectively eliminated by CD138.CAR-Ts, strengthening the rationale that CD138.CAR-Ts will be effective in eliminating the reservoir of MM cancer stem cells.

Taking into consideration the observed mild toxicity associated with the anti-CD138 antibody-drug conjugate (BT062) and the potential amplification of these toxic effects in the setting of CAR-T therapy, we have assessed the reactivity of CD138.CAR-Ts against human primary endothelial and epithelial cells, and observed no reactivity by CD138.CAR-Ts. Furthermore, because diarrhea was noted in patients treated with the BT062 antibody [17–19], we also explored the potential GI toxicity of targeting CD138 by evaluating the *in vitro* activity of CD138.CAR-Ts against GI stem cells (both undifferentiated and differentiated), and again observed no toxicity. Based on these data a new investigational new drug (IND) has been prepared to target CD138 with autologous CD138.CAR-Ts in patients with relapsed/refractory MM, and the study is currently open for enrollment (ClinicalTrials.gov Identifier: NCT03672318).

In conclusion, we provided efficacy and safety data supporting the clinical assessment of CD138.CAR-Ts in patients with MM. If safe and effective, CD138 could be combined with BCMA in an attempt to eradicate MM. In addition, to prevent antigen escape, future research should also take in consideration the microenvironment of MM that is enriched in immunosuppressive cells, cytokines and other soluble factors, like TGF-β or kynurenines that can potentially inhibit the antitumor activity of CAR-Ts [42]. Furthermore, the MM niche is highly hypoxic and hypoglycemic, and may limit the long-term efficacy of CAR-Ts [43]. All these elements will need to be clearly identified so that strategies to overcome such challenges can be successfully developed.

## MATERIALS AND METHODS

### Cell lines and human samples

The CD138^+^ MM-derived cell lines OPM-2, RPMI-8226, U266-B1, MMS1 and the CD138^−^ cell line Raji were obtained from the American Type Culture Collection (ATCC, Rockville, MD, USA). All cells were maintained in culture with RPMI-1640 medium (Hyclone, Logan, Utah) containing 10% fetal bovine serum (FBS, Hyclone) and 2 mM L-glutamine (GIBCO-BRL, Gaithersburg, MD). Coronary, pulmonary and dermal endothelial cells and colon epithelial cells were purchased from ATCC and grown in the recommended media, following manufacturer's instruction. Cells were kept in culture for less than 6 consecutive months, after which aliquots from the original expanded vial were used. Cells were maintained in a humidified atmosphere containing 5% CO_2_ at 37° C. Tumor cell lines were routinely tested to exclude contamination with mycoplasma and assessed for the expression of CD138 to confirm identity. Buffy coats from healthy volunteer blood donors were obtained through the Gulf Coast Regional Blood Center, Houston, TX. Peripheral blood (PB) and bone marrow (BM) samples from de-identified patients with MM were collected according to local institutional review board (IRB) approved protocols (Baylor College of Medicine, Houston, TX). CD138^+^ cells from BM were isolated by immunomagnetic selection using paramagnetic beads specific for CD138 (Miltenyi), following manufacturer's recommendation. In selected experiments, cells were treated with diluent or melphalan, dexamethasone, fludarabine or cytoxan for up to 72 hrs.

### Retroviral vectors

The CD138-specific single-chain variable fragment (scFv) was obtained from the human murine chimeric antibody specific for the human CD138 antigen (BT062) (patent US20090175863). The sequence of the scFv was codon optimized to enhance its translation in mammalian cells and cloned into CAR molecules [44]. All CAR constructs were subcloned into the SFG retroviral backbone. To produce the retroviral supernatant, 293T cells were co-transfected with Peg-Pam-e plasmid containing the sequence for MoMLV gag-pol, and the RDF plasmid containing the sequence for the RD114 envelope, using the Fugene6 transfection reagent (Roche Indianapolis, IN) as previously described [44]. The generation of retrovirus vectors encoding Firefly Luciferase gene (FFLuc) or the fusion protein eGFP-Firefly Luciferase (eGFP-FFLuc) have been previously described [44]. The FFluc specific vector, which also contains the puromycin resistance gene, was used for stable transduction of tumor cell lines. For GMP translational and validation studies a clinical grade packaging cell lines was generated by using the PG13 packaging cell line cells (gibbon ape leukemia virus pseudotyping packaging cell line; CRL-10686, ATCC) as previously described [13]. We used the highest-titer clone to establish the master cell bank as recommended by FDA guidelines.

### Generation of CD138.CAR activated T cells

Peripheral blood mononuclear cells (PBMCs) were isolated from 7 healthy volunteer blood donors and from 6 MM patients by Lymphoprep (Accurate Chemical and Scientific Corp., Westbury, NY) density gradient centrifugation. Activated T cells were generated using immobilized CD3 and CD28 antibodies (Miltenyi), and transduced with CD138.CARs on RetroNectin (FN CH-296; Takara, Otsu, Japan)-coated 24-well plate [44]. CD138.CAR transduced T cells (CD138.CAR-Ts) were then expanded in complete media (RPMI1640 [Hyclone] 45%, Click's medium [Irvine Scientific, Santa Ana, CA] 45%, supplemented with 10% FBS and L-glutamine) containing recombinant human interleukin-2 (rhIL-2, 100U/mL; Proleukine; Chiron, Emeryville, CA) or IL-7 (10ng/ml; Peprotech, Rocky Hill, NJ) and IL-15 (5ng/ml; Peprotech), every 2–3 days as previously described [45]. As negative controls, T cells transduced with an empty vector were grown in parallel (control T cells, Ctr-Ts).

### Immunophenotype

We used phycoerythrin, fluorescein isothiocyanate, peridinin chlorophyll protein, allophycocyanin, Alexa-750, Alexa-700, Phycoerythrin Cyanin 7, Pacific Blue, or Krome-Orange-conjugated CD3, CD4, CD8, CD19, CD45RA, CD45RO, CCR7, CD138, CD31, CD80, CD86, CD83, CD40L, CD137L, OX40L, PD-L1 (Becton-Dickinson-Pharmingen, San Diego, CA and Beckman coulter) monoclonal antibodies (Abs). We included control samples labeled with appropriate isotype Ab and used a “fluorescence minus one” strategy for multicolor staining. We used the Alexa Fluor 647-labeled AffiniPure F(ab')_₂_ Fragment Goat Anti-Mouse IgG, F(ab')_₂_ fragment specific (Jackson ImmunoResearch, West Grove, PA) to detect CD138.CAR expression in T cells. We analyzed cells using a FACScan (Becton-Dickinson) equipped with a filter set for 4 fluorescence signals, using CellQuest software, or a FACS-Canto II, using DIVA software. In some cases cells were collected using the Gallios Flow Cytometer (Beckman Coulter, Indianapolis, IN) and analyzed using FlowJo software version 9.3 (Tree Star, Ashland, OR) or Kaluza software (Beckman Coulter).

### Gastrointestinal stem cell differentiation and CAR-T co-culture

Adult duodenum stem cells and murine feeder cells were purchased from R&D (Minneapolis, MN). The expansion and differentiation of adult GI stem cell was performed following manufacturer's instructions (MimEX GI kit, R&D). Undifferentiated and differentiated stem cells were then co-cultured with 5 × 10^5^ Ctr-Ts, CD19.CAR-Ts or CD138.CAR-Ts, and 24 hours later supernatants were collected and the production of IFNγ was analyzed by using ELISA kit (R&D). The CD19.CAR was included to further ensure lack of activity due to a non-specific scFv.

### Hoechst SP cell staining

For Hoechst SP analysis, MM tumor cell lines and primary MM tumor cells were resuspended at 1×10^6^ cells/ml in pre-warmed media with 2% FBS and 10 mM HEPES (Sigma-Aldrich, St. Louis, MO). Hoechst 33342 dye (Sigma) was added to a final concentration of 5 μg/ml as previously described [22–24]. Cells were then incubated at 37° C for 105 minutes, washed in ice cold Hank's buffered salt solution supplemented with 2% FBS and 10 mM HEPES (HBSS^+^) and then immediately placed on ice. For analysis of the SP cell frequency, a live gate was defined by using the Hoechst red and blue axes to exclude dead cells (PI positive) and debris. For further phenotypic analyses, Hoechst labeled cells were subsequently stained with fluorescent-conjugated CD3 and CD138 mAbs for 15 minutes on ice. Cells were then washed again and resuspended in ice cold HBSS^+^ buffer containing 2 μg/ml propidium iodide (PI; Sigma-Aldrich) for dead cell exclusion. Gates for SP and NSP cells were subsequently used to determine the expression of surface antigens using a BD LSRII analyzer (BD Bioscience). For the isolation of SP and NSP cells, we sorted them using a high-speed cell sorter (MoFlo, Dako Cytomation, Fort Collins, CO).

### Evaluation of CAR-T cells function

To assess short-term cytotoxic activity we used a standard ^51^Cr release assay [44]. Chromum-51 labeled CD138^+^ and CD138^−^ target cells were incubated with T cells at different effector to target ratios, and in medium alone or in 1% Triton X-100 (Sigma-Aldrich) to determine spontaneous and maximum ^51^Cr-release, respectively. The mean percentage of specific lysis of triplicate wells was calculated as follows: [(test counts − spontaneous counts)/(maximum counts − spontaneous counts)] × 100%. Coculture experiments were also performed. Control and CD138.CAR-Ts were cocultured in 24-well plates in the presence of CD138^+^ or CD138^−^ target cells (at 1:1 E:T ratio) and in the absence of exogenous cytokines. Phenotypic analyses were performed on day 3 or 5 of coculture and T cells and tumor cells detected using CD3, CD138, or CD19 Abs. We also cocultured T cells with autologous primary MM cells and with endothelial or epithelial cells. In the latter case, CD31 Ab was used to identify endothelial cells. Co-culture supernatant was harvested after 24 hours of culture and cytokines measured using specific cytometric bead arrays (BD), or specific ELISAs (R&D System) according to manufacturer's instructions. The soluble CD138 was also assessed in the culture supernatant of tumor cells using a specific ELISA kit (R&D System).

### Quantitative real-time PCR

To compare the mRNA levels of CD138 in different normal and multiple myeloma cell lines, > 1 × 10^6^ cells were collected. Total RNA was isolated with RNeasy plus mini kit (Qiagen, Valencia, CA) and cDNA was synthesized with SuperScript VILO cDNA Synthesis Kit (Thermo Fisher) per manufacturer's instructions. qRT-PCR was performed on Quanta Studio 6 Flex (Applied Biosystem) with primers for CD138 (Taqman, Thermo Fisher) and β-actin (Taqman, Thermo Fisher) was used as an internal control. Each sample was performed in triplets and the relative amount of mRNA was quantified by using comparative Ct method.

### Xenogeneic mouse models

To evaluate the activity of CD138.CAR-Ts *in vivo*, 6–8 wks old male and female NSG (NOD.Cg-Prkdc Il2rg/SzJ) mice were injected intravenously (i.v.) with the CD138^+^ tumor cell line OPM-2 labeled with FFLuc gene (4 × 10^6^/mouse). Ten days later, mice received i.v. via tail vain either Ctr-Ts or CD138.CAR-Ts (1 × 10^7^/mouse). Tumor growth was monitored weekly by injecting mice intraperitoneally (i.p.) with D-luciferin (150 mg/kg). Photon emission was analyzed using the Xenogen-IVIS Imaging System as previously validated [44].

### Statistical analysis

All *in vitro* data are presented as mean ± SEM, unless indicated. Student's *t*-test was used to determine the statistical significance of differences between samples, and *P* < .05 was accepted as indicating a significant difference. For the bioluminescent experiments, intensity signals were log-transformed and summarized using mean ± SD at baseline and multiple subsequent time points for each group of mice. Changes in intensity of signal from baseline at each time point were calculated and compared using paired *t*-tests or Wilcoxon signed-ranks test.

## SUPPLEMENTARY MATERIALS FIGURES



## Abbreviations

MM: Multiple myeloma
CAR: chimeric antigen receptor
BCMA: B-cell maturation antigen
IHC: immunohistochemetry
Ctr: control

## References

[1] Palumbo A, Anderson K (2011). Multiple myeloma. N Engl J Med.

[2] Moreau P, San Miguel J, Sonneveld P, Mateos MV, Zamagni E, Avet-Loiseau H, Hajek R, Dimopoulos MA, Ludwig H, Einsele H, Zweegman S, Facon T, Cavo M, ESMO Guidelines Committee (2017). Multiple myeloma: ESMO Clinical Practice Guidelines for diagnosis, treatment and follow-up. Ann Oncol.

[3] Siegel RL, Miller KD, Jemal A (2018). Cancer statistics, 2018. CA Cancer J Clin.

[4] Dosani T, Carlsten M, Maric I, Landgren O (2015). The cellular immune system in myelomagenesis: NK cells and T cells in the development of MM and their uses in immunotherapies. Blood Cancer J.

[5] Alyea E, Weller E, Schlossman R, Canning C, Mauch P, Ng A, Fisher D, Gribben J, Freeman A, Parikh B, Richardson P, Soiffer R, Ritz J, Anderson KC (2003). Outcome after autologous and allogeneic stem cell transplantation for patients with multiple myeloma: impact of graft-versus-myeloma effect. Bone Marrow Transplant.

[6] Lokhorst HM, Plesner T, Laubach JP, Nahi H, Gimsing P, Hansson M, Minnema MC, Lassen U, Krejcik J, Palumbo A, van de Donk NW, Ahmadi T, Khan I (2015). Targeting CD38 with Daratumumab Monotherapy in Multiple Myeloma. N Engl J Med.

[7] Lonial S, Dimopoulos M, Palumbo A, White D, Grosicki S, Spicka I, Walter-Croneck A, Moreau P, Mateos MV, Magen H, Belch A, Reece D, Beksac M, ELOQUENT-2 Investigators (2015). Elotuzumab therapy for relapsed or refractory multiple myeloma. N Engl J Med.

[8] Rajkumar SV, Kyle RA (2016). Progress in Myeloma - A monoclonal breakthrough. N Engl J Med.

[9] Joshua D, Suen H, Brown R, Bryant C, Ho PJ, Hart D, Gibson J (2016). The T cell in myeloma. Clin Lymphoma Myeloma Leuk.

[10] Guillerey C, Harjunpää H, Carrié N, Kassem S, Teo T, Miles K, Krumeich S, Weulersse M, Cuisinier M, Stannard K, Yu Y, Minnie SA, Hill GR (2018). TIGIT immune checkpoint blockade restores CD8+ T-cell immunity against multiple myeloma. Blood.

[11] Minnie SA, Kuns RD, Gartlan KH, Zhang P, Wilkinson AN, Samson L, Guillerey C, Engwerda C, MacDonald KP, Smyth MJ, Markey KA, Vuckovic S, Hill GR (2018). Myeloma escape after stem cell transplantation is a consequence of T-cell exhaustion and is prevented by TIGIT blockade. Blood.

[12] Li Y, Chen S, Yang L, Chen S, Lin C, Wang L, Lu Y, Geng S, Du X, Schmidt CA (2011). Change in expression pattern of TCR-CD3 complex in patients with multiple myeloma. Hematology.

[13] Ramos CA, Savoldo B, Torrano V, Ballard B, Zhang H, Dakhova O, Liu E, Carrum G, Kamble RT, Gee AP, Mei Z, Wu MF, Liu H (2016). Clinical responses with T lymphocytes targeting malignancy-associated κ light chains. J Clin Invest.

[14] Brudno JN, Maric I, Hartman SD, Rose JJ, Wang M, Lam N, Stetler-Stevenson M, Salem D, Yuan C, Pavletic S, Kanakry JA, Ali SA, Mikkilineni L (2018). T cells genetically modified to express an Anti-B-Cell maturation antigen chimeric antigen receptor cause remissions of poor-prognosis relapsed multiple myeloma. J Clin Oncol.

[15] Palaiologou M, Delladetsima I, Tiniakos D (2014). CD138 (syndecan-1) expression in health and disease. Histol Histopathol.

[16] Moreaux J, Sprynski AC, Dillon SR, Mahtouk K, Jourdan M, Ythier A, Moine P, Robert N, Jourdan E, Rossi JF, Klein B (2009). APRIL and TACI interact with syndecan-1 on the surface of multiple myeloma cells to form an essential survival loop. Eur J Haematol.

[17] Heffner LT, Jagannath S, Zimmerman TM, Lee KP, Rosenblatt J, Lonial S, Lutz RJ, Czeloth N, Osterroth F, Ruehle M, Beelitz MA, Wartenberg-Demand A, Haeder T (2012). BT062, an Antibody-Drug Conjugate Directed Against CD138, Given Weekly for 3 Weeks in Each 4 Week Cycle: Safety and Further Evidence of Clinical Activity. Blood.

[18] Kelly KR, Chanan-Khan A, Heffner LT, Somlo G, Siegel DS, Zimmerman T, Karnad A, Munshi NC, Jagannath S, Greenberg AL, Lonial S, Roy V, Ailawadhi S (2014). Indatuximab ravtansine (BT062) in combination with lenalidomide and low-dose dexamethasone in patients with relapsed and/or refractory multiple myeloma: clinical activity in patients already exposed to lenalidomide and bortezomib. Blood.

[19] Kelly KR, Siegel DS, Chanan-Khan AA, Somlo G, Heffner LT, Jagannath S, Zimmerman T, Munshi NC, Madan S, Mohrbacher A, Lonial S, Barmaki-Rad F, Rühle M (2016). Indatuximab Ravtansine (BT062) in Combination with Low-Dose Dexamethasone and Lenalidomide or Pomalidomide: Clinical Activity in Patients with Relapsed / Refractory Multiple Myeloma. Blood.

[20] Guo B, Chen M, Han Q, Hui F, Dai H, Zhang W, Zhang Y, Wang Y, Zhu H, Han W (2016). CD138-directed adoptive immunotherapy of chimeric antigen receptor (CAR)-modified T cells for multiple myeloma. Journal of Cellular Immunotherapy.

[21] Tian C, Yang H, Zhu L, Zhang Q, Cao Z, Zhang Y (2017). Anti-CD138 chimeric antigen receptor-modified T cell therapy for multiple myeloma with extensive extramedullary involvement. Ann Hematol.

[22] Goodell MA, Rosenzweig M, Kim H, Marks DF, DeMaria M, Paradis G, Grupp SA, Sieff CA, Mulligan RC, Johnson RP (1997). Dye efflux studies suggest that hematopoietic stem cells expressing low or undetectable levels of CD34 antigen exist in multiple species. Nat Med.

[23] Hirschmann-Jax C, Foster AE, Wulf GG, Nuchtern JG, Jax TW, Gobel U, Goodell MA, Brenner MK (2004). A distinct “side population” of cells with high drug efflux capacity in human tumor cells. Proc Natl Acad Sci U S A.

[24] Hong LK, Chen Y, Smith CC, Montgomery SA, Vincent BG, Dotti G, Savoldo B (2018). CD30-Redirected Chimeric Antigen Receptor T Cells Target CD30+ and CD30- Embryonal Carcinoma via Antigen-Dependent and Fas/FasL Interactions. Cancer Immunol Res.

[25] Hirschmann-Jax C, Foster AE, Wulf GG, Goodell MA, Brenner MK (2005). A distinct “side population” of cells in human tumor cells: implications for tumor biology and therapy. Cell Cycle.

[26] Mikkilineni L, Kochenderfer JN (2017). Chimeric antigen receptor T-cell therapies for multiple myeloma. Blood.

[27] Drent E, Groen RW, Noort WA, Themeli M, Lammerts van Bueren JJ, Parren PW, Kuball J, Sebestyen Z, Yuan H, de Bruijn J, van de Donk NW, Martens AC, Lokhorst HM, Mutis T (2016). Pre-clinical evaluation of CD38 chimeric antigen receptor engineered T cells for the treatment of multiple myeloma. Haematologica.

[28] Drent E, Themeli M, Poels R, de Jong-Korlaar R, Yuan H, de Bruijn J, Martens ACM, Zweegman S, van de Donk NWCJ, Groen RWJ, Lokhorst HM, Mutis T (2017). A Rational Strategy for Reducing On-Target Off-Tumor Effects of CD38-Chimeric Antigen Receptors by Affinity Optimization. Mol Ther.

[29] Hsi ED, Steinle R, Balasa B, Szmania S, Draksharapu A, Shum BP, Huseni M, Powers D, Nanisetti A, Zhang Y, Rice AG, van Abbema A, Wong M (2008). CS1, a potential new therapeutic antibody target for the treatment of multiple myeloma. Clin Cancer Res.

[30] Chu J, He S, Deng Y, Zhang J, Peng Y, Hughes T, Yi L, Kwon CH, Wang QE, Devine SM, He X, Bai XF, Hofmeister CC, Yu J (2014). Genetic modification of T cells redirected toward CS1 enhances eradication of myeloma cells. Clin Cancer Res.

[31] Liebisch P, Eppinger S, Schöpflin C, Stehle G, Munzert G, Döhner H, Schmid M (2005). CD44v6, a target for novel antibody treatment approaches, is frequently expressed in multiple myeloma and associated with deletion of chromosome arm 13q. Haematologica.

[32] Casucci M, Nicolis di Robilant B, Falcone L, Camisa B, Norelli M, Genovese P, Gentner B, Gullotta F, Ponzoni M, Bernardi M, Marcatti M, Saudemont A, Bordignon C (2013). CD44v6-targeted T cells mediate potent antitumor effects against acute myeloid leukemia and multiple myeloma. Blood.

[33] McEarchern JA, Smith LM, McDonagh CF, Klussman K, Gordon KA, Morris-Tilden CA, Duniho S, Ryan M, Boursalian TE, Carter PJ, Grewal IS, Law CL (2008). Preclinical characterization of SGN-70, a humanized antibody directed against CD70. Clin Cancer Res.

[34] Shaffer DR, Savoldo B, Yi Z, Chow KK, Kakarla S, Spencer DM, Dotti G, Wu MF, Liu H, Kenney S, Gottschalk S (2011). T cells redirected against CD70 for the immunotherapy of CD70-positive malignancies. Blood.

[35] Wang QJ, Yu Z, Hanada KI, Patel K, Kleiner D, Restifo NP, Yang JC (2017). Preclinical Evaluation of Chimeric Antigen Receptors Targeting CD70-Expressing Cancers. Clin Cancer Res.

[36] Lee L, Draper B, Chaplin N, Philip B, Chin M, Galas-Filipowicz D, Onuoha S, Thomas S, Baldan V, Bughda R, Maciocia P, Kokalaki E, Neves MP (2018). An APRIL-based chimeric antigen receptor for dual targeting of BCMA and TACI in multiple myeloma. Blood.

[37] Savoldo B, Rooney CM, Di Stasi A, Abken H, Hombach A, Foster AE, Zhang L, Heslop HE, Brenner MK, Dotti G (2007). Epstein Barr virus specific cytotoxic T lymphocytes expressing the anti-CD30zeta artificial chimeric T-cell receptor for immunotherapy of Hodgkin disease. Blood.

[38] Hombach A, Heuser C, Sircar R, Tillmann T, Diehl V, Pohl C, Abken H (1998). An anti-CD30 chimeric receptor that mediates CD3-zeta-independent T-cell activation against Hodgkin's lymphoma cells in the presence of soluble CD30. Cancer Res.

[39] Ghosh N, Matsui W (2009). Cancer stem cells in multiple myeloma. Cancer Lett.

[40] Garfall AL, Maus MV, Hwang WT, Lacey SF, Mahnke YD, Melenhorst JJ, Zheng Z, Vogl DT, Cohen AD, Weiss BM, Dengel K, Kerr ND, Bagg A (2015). Chimeric Antigen Receptor T Cells against CD19 for Multiple Myeloma. N Engl J Med.

[41] Garfall AL, Stadtmauer EA, Hwang WT, Lacey SF, Melenhorst JJ, Krevvata M, Carroll MP, Matsui WH, Wang Q, Dhodapkar MV, Dhodapkar K, Das R, Vogl DT (2018). Anti-CD19 CAR T cells with high-dose melphalan and autologous stem cell transplantation for refractory multiple myeloma. JCI Insight.

[42] Sun C, Dotti G, Savoldo B (2016). Utilizing cell-based therapeutics to overcome immune evasion in hematologic malignancies. Blood.

[43] Xu Y, Chaudhury A, Zhang M, Savoldo B, Metelitsa LS, Rodgers J, Yustein JT, Neilson JR, Dotti G (2016). Glycolysis determines dichotomous regulation of T cell subsets in hypoxia. J Clin Invest.

[44] Diaconu I, Ballard B, Zhang M, Chen Y, West J, Dotti G, Savoldo B (2017). Inducible Caspase-9 Selectively Modulates the Toxicities of CD19-Specific Chimeric Antigen Receptor-Modified T Cells. Mol Ther.

[45] Xu Y, Zhang M, Ramos CA, Durett A, Liu E, Dakhova O, Liu H, Creighton CJ, Gee AP, Heslop HE, Rooney CM, Savoldo B, Dotti G (2014). Closely related T-memory stem cells correlate with in vivo expansion of CAR.CD19-T cells and are preserved by IL-7 and IL-15. Blood.

