# Supplementary material for: Safety and efficacy of targeting CD138 with a chimeric antigen receptor for the treatment of multiple myeloma
Source: Oncotarget. 2019 Mar 22;10(24):2369–83. doi: 10.18632/oncotarget.26792 (PMC6481321; doi:10.18632/oncotarget.26792)
Supplement: Supplementary file 1 [file oncotarget-10-2369-s001.pdf]

Safety and efficacy of targeting CD138 with a chimeric antigen receptor for the treatment of multiple myeloma

SUPPLEMENTARY MATERIALS

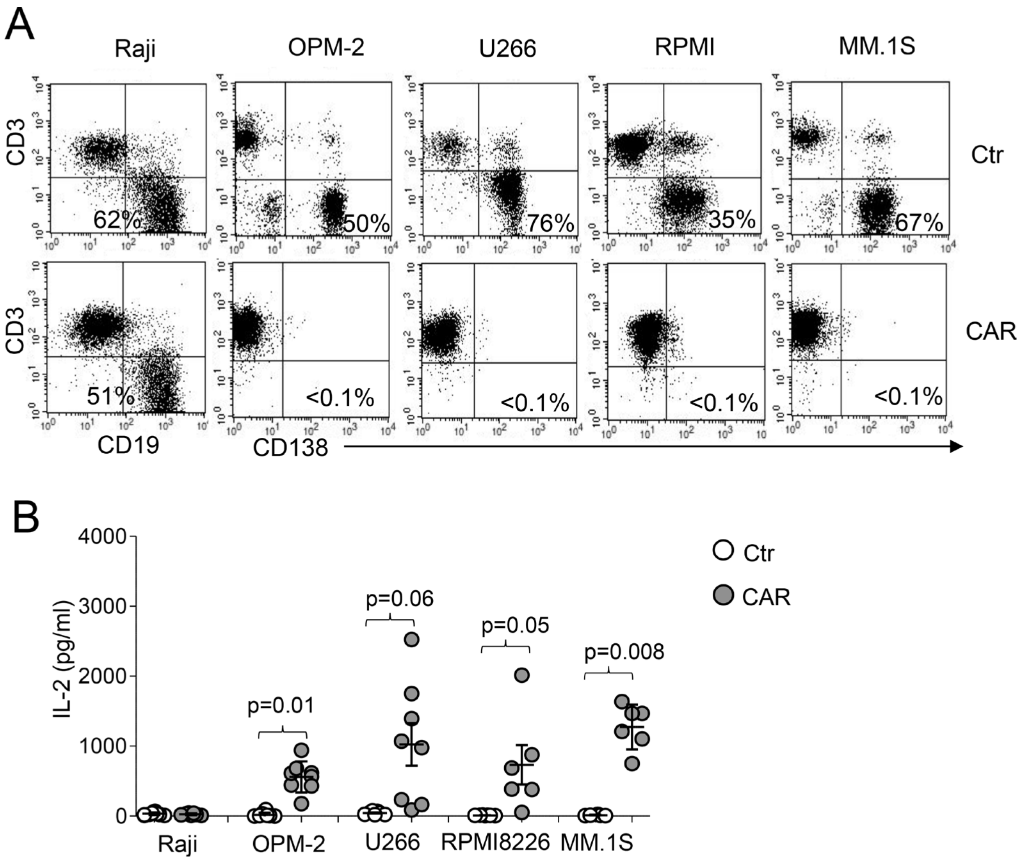

Supplementary Figure 1: Function of CD138.CAR-Ts against CD138[+] target cells. (**A**) Representative flow plots for experiments of co-culture of Ctr-Ts (upper panels) and CD138.CAR-Ts (CAR1, lower panels) with MM cell lines. (**B**) Quantification of IL-2 in sup of Ctr-Ts vs. CD138.CAR-Ts co-cultured for 24 hrs with four CD138[+] cells (OPM-2, U266, RPMI, MM.1S cells), at 1:1 ratio and quantified by Bioplex assays (shown are *p* value, paired *t*-test). CD138.CAR are from 1 - 3 donors for each CARs.

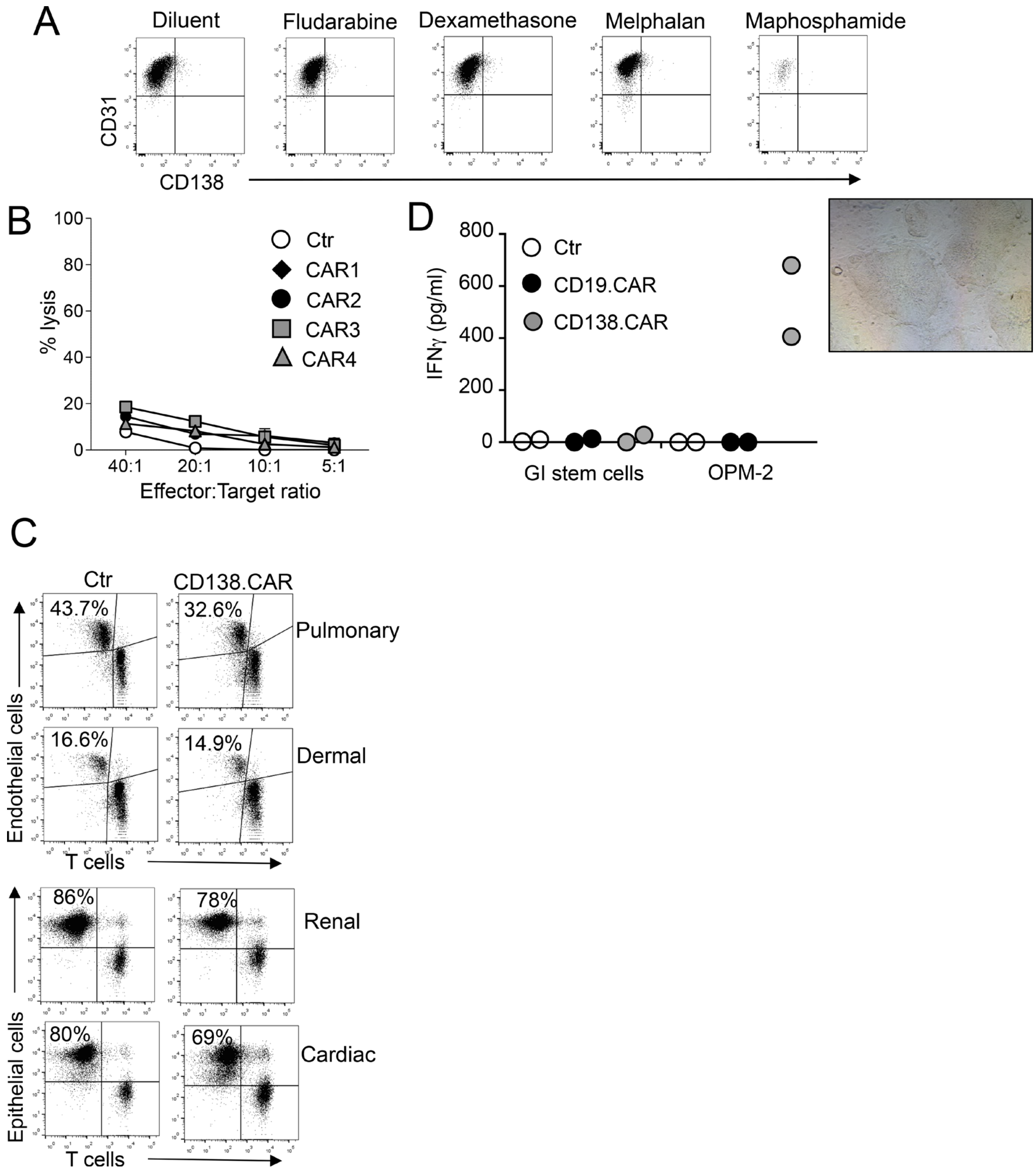

Supplementary Figure 2: Safety of CD138.CAR-Ts. (**A**) Evaluation of the expression of CD31 (endothelial marker) and CD138 by endothelial cells (shown are pulmonary cells) in the presence of diluent or drugs. Cells treated with mafosfamide had reduced viability. (**B**) Results of a standard ^{51}Cr release assay with pulmonary cells and CAR-Ts at the indicted T cell to target cell ratio. (**C**) Representative flow plots of Ctr-Ts or CD138.CAR-Ts with the indicated endothelial (identified by CD31) or epithelial (identified by CD276) cells co-cultured with Ctr-Ts or CD138.CAR-Ts (identified by CD3) Shown is CAR2 as representative example. (**D**) Left graph shows the IFNγ production by Ctr-Ts, CD19.CAR-Ts or CD138.CAR-Ts (CAR3) co-cultured with undifferentiated and spontaneously differentiated adult GI stem cells. OPM-2 cells were used as positive control. Right image shows undifferentiated adult GI stem cells.

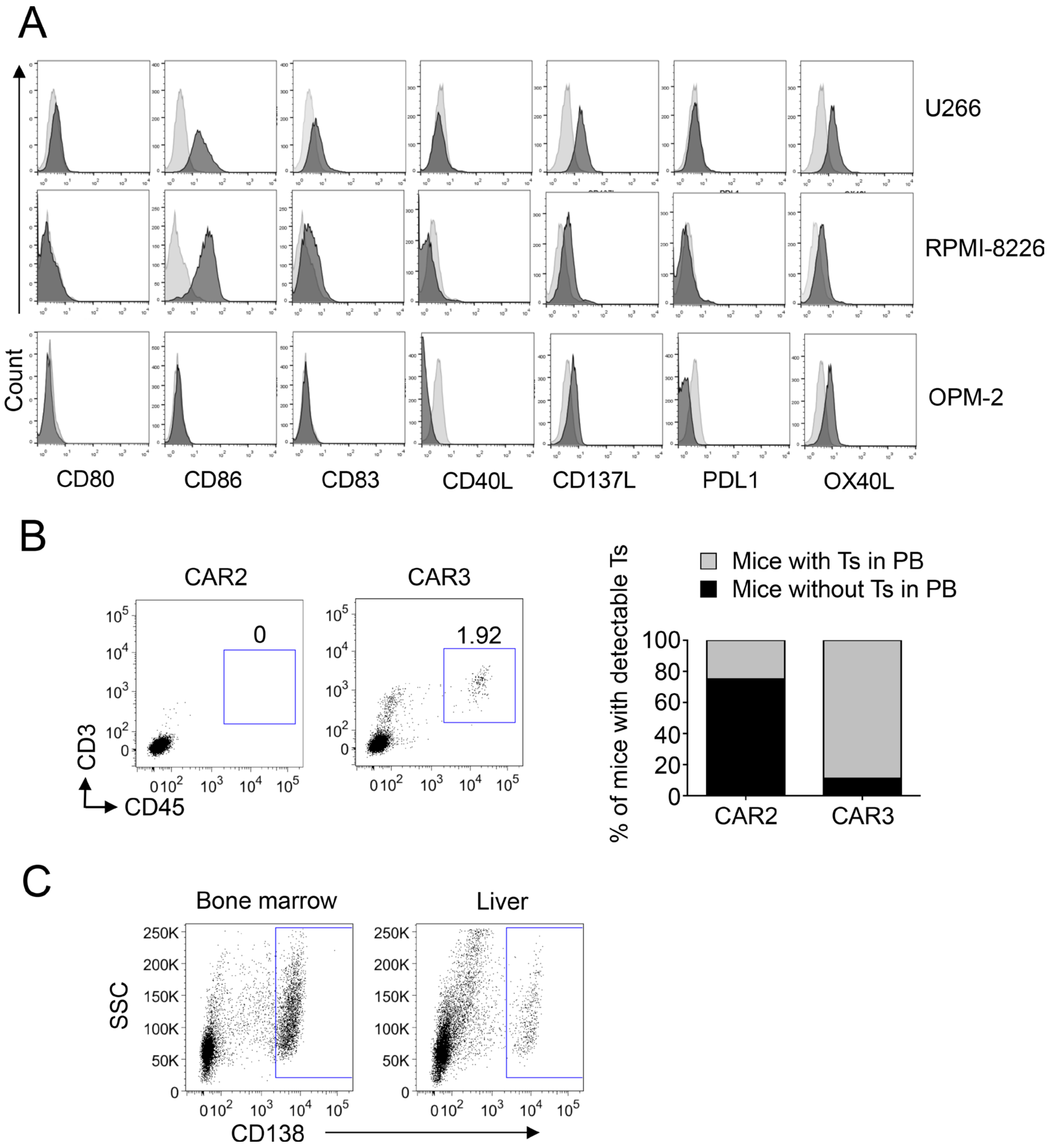

Supplementary Figure 3: Characterization of MM tumor cell lines. (**A**) Flow plots showing the expression of the indicated molecules in 3 MM cell lines, based on flow cytometry. Light grays lines represent staining of cells with appropriate isotype control (all phycoerythrin). (**B**) The analysis of the peripheral blood (PB) of mice received CAR2-Ts or CAR3-Ts at 85-90 days post tumor injection. Peripheral blood was collected and analyzed by flow cytometry for the presence of circulating T cells (hCD45+, hCD3+). Left panel, flow plots for 1 representative mice from each group; right panel, summary of mice with detectable T cells in the PB. (**C**) Flow plots showing expression of CD138 in OPM-2 cells isolated from mice treated with CD138.CAR-Ts and showing tumor recurrence after treatment.

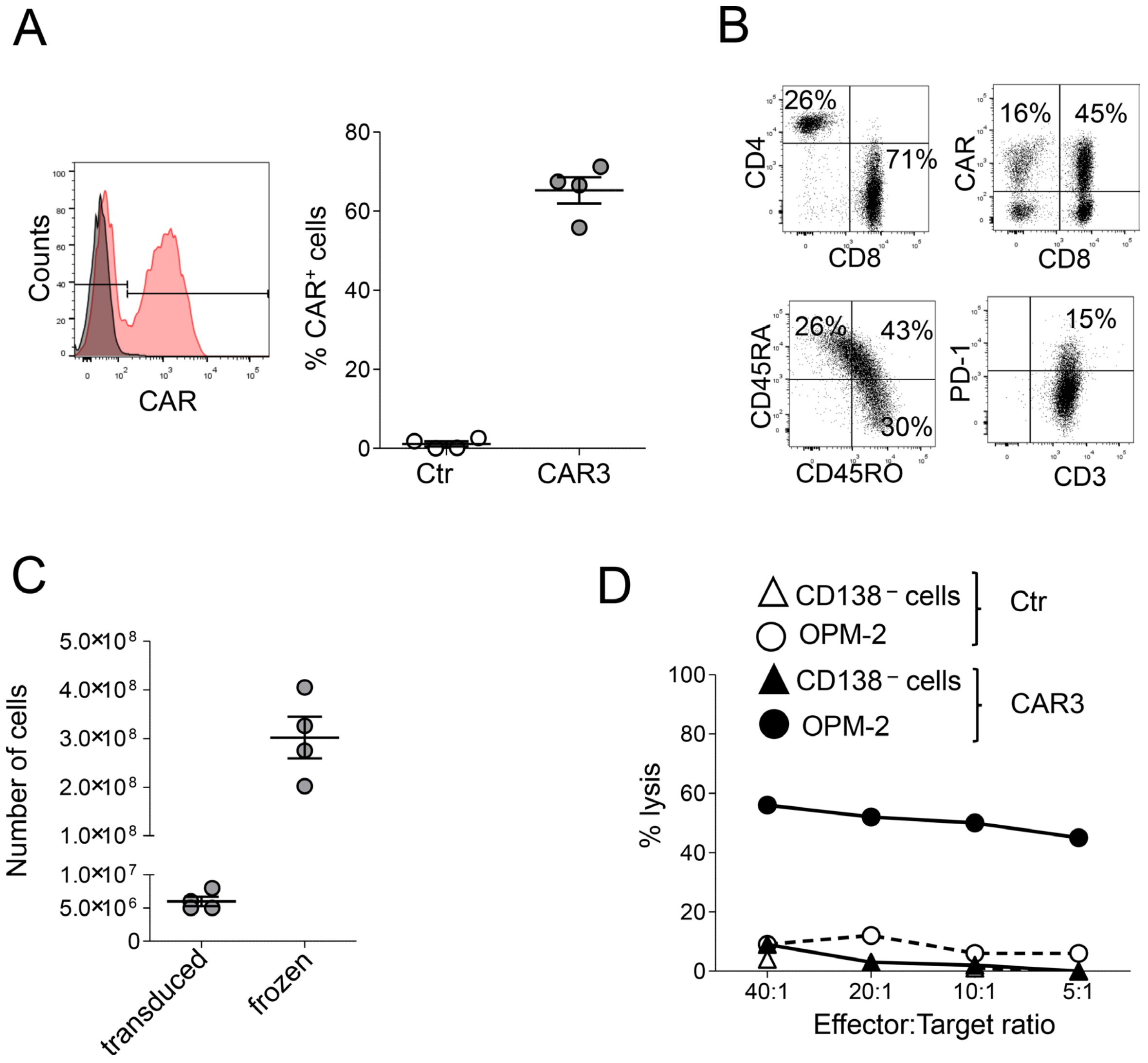

Supplementary Figure 4: Scale up of the manufacturing of CD138.CAR-Ts. (**A**) Flow plots showing CD138.CAR (CAR3) expression by T cells transduced with retroviral supernatant obtained from the MCB released for clinical use (*n* = 4). (**B**) Phenotypic characterization of CD138.CAR-Ts using flow cytometry for 1 representative donor. (**C**) Cell expansion of CD138.CAR-Ts by day 12 post transduction (*n* = 4). (**D**) Cytotoxic activity of CD138.CAR-Ts against CD138+ or CD138– tumor cells in a standard ^{51}Cr release assays for 1 representative donor.
